# Genomes of *Escherichia coli* bacteraemia isolates originating from urinary tract foci contain more virulence-associated genes than those from non-urinary foci and neutropaenic hosts

**DOI:** 10.1016/j.jinf.2018.10.011

**Published:** 2018-12

**Authors:** Adam P. Dale, Anish K. Pandey, Richard J. Hesp, Konstantinos Belogiannis, Jay R. Laver, Clifford C. Shone, Robert C. Read

**Affiliations:** a Academic Unit of Clinical and Experimental Sciences, Faculty of Medicine, University of Southampton, LC71A, C Level, South Academic Block, Southampton General Hospital, Southampton SO16 6YD, UK; b National Infection Service, Public Health England, Porton Down, Salisbury SP4 0JG, UK; c NIHR Southampton Biomedical Research Centre, University Hospital Southampton, Southampton SO16 6YD, UK

**Keywords:** *Escherichia coli*, ExPEC, Bacteraemia, Virulence factor, Neutropaenia, Antimicrobial resistance, Whole genome sequencing

## Abstract

**Objectives:**

*Escherichia coli* is the leading cause of bacteraemia. In an era of emerging multi-drug-resistant strains, development of effective preventative strategies will be informed by knowledge of strain diversity associated with specific infective syndromes/patient groups. We hypothesised that the number of virulence factor (VF) genes amongst bacteraemia isolates from neutropaenic patients would be lower than isolates from immunocompetent patients.

**Methods:**

Immunocompetent and neutropaenic adults with *E. coli* bacteraemia were recruited prospectively and the source of bacteraemia determined. VF gene profiles were established *in silico* following whole genome sequencing.

**Results:**

Isolates from individual patients were monoclonal. Strains from immunocompetent patients with urinary tract infective foci (UTIF) harboured more VF genes (median number of VF genes 16, range 8–24) than isolates from both immunocompetent patients with non-UTIF (10, 2–22, *p* = 0.0058) and neutropaenic patients with unknown focus of infection (NPUFI) (8, 3–13, *p* < 0.0001). Number of VF genes (OR 1.21, 95% CIs 1.01–1.46, *p* = 0.039) and urinary catheter/recurrent urinary tract infection (OR 12.82, 95% CIs 1.24–132.65, *p* = 0.032) were independent predictors of bacteraemia secondary to UTIF vs. non-UTIF in immunocompetent patients. *papA, papC, papE/F, papG, agn43, tia, iut, fyuA, kpsM* and *sat* were significantly more prevalent amongst UTIF- vs non-UTIF-originating isolates amongst immunocompetent patients, while *papC, papE/F, papG, agn43, tia, fyuA, hlyA, usp* and *clb* were significantly more prevalent amongst UTIF- vs NPUFI-associated isolates.

**Conclusions:**

Bacteraemia-associated *E. coli* strains originating from UTIF have distinct VF gene profiles from strains associated with non-UTIF- and NPUFI. This diversity must be addressed in the design of future vaccines to ensure adequate coverage of strains responsible for site-specific disease.

## Introduction

Extra-intestinal pathogenic *E. coli* (ExPEC) are the leading cause of bacteraemia world-wide and are associated with urinary tract, hepatobiliary/gastro-intestinal tract, skin/soft tissue and respiratory tract infections, as well as neonatal meningitis and febrile neutropaenia.^1^ The scale of the ExPEC problem is large,^2^ particularly in the context of increasing antimicrobial resistance and the current dominance of multi-drug-resistant (MDR) sequence types (STs), e.g. ST 131.^3^

ExPEC possess multiple virulence factor (VF) genes encoding adhesins, iron-acquisition systems, protectins/invasins, and toxins, and are gut colonisers in >10% of individuals.^4^ ExPEC have previously been defined as those that contain at least two of the following VF-encoding genes: *papA* and/or *papC* (P fimbriae), *sfa/foc* (S fimbriae), *afa/draBC* (Dr binding adhesins), *kpsM II* (group 2 capsule) and *iutA* (aerobactin receptor).^5^ ExPEC VFs (herein referred to as VFs) have been associated with site-specific disease, e.g. pyelonephritis (*pap, afa/draBC* and *sfa* adhesin genes*, iha* adhesion siderophore gene, and the *ibeA* protectin invasin gene)^1^, ^6^ and neonatal meningitis (*kps* capsule gene, *ompA* and *ibe* protectin/invasin genes*, fimH* adhesin gene, and *cnf1* toxin gene).^1^, ^7^ A broad range of STs can cause disease but 50–70% of disease-associated isolates belong to STs 69, 73, 95, 127 and 131.^8^

In severely immunocompromised patients, e.g. those with haematological malignancy and neutropaenia, *E. coli* bacteraemia often occurs in the absence of any clinically-identifiable focus as a consequence of direct translocation from the gut.^9^ This process likely occurs secondary to damage to the structural integrity of the intestinal mucosa, as a result of compromised mucosal/systemic immunity, or due to bacterial overgrowth.^10^ The contribution of VFs in this context is undefined.

We hypothesised that, in severe immunocompromise, *E. coli* strains with fewer VFs would be able to translocate across the bowel and survive haematogenously compared with bacteraemia strains from immunocompetent patients. Additionally, we posited that *E. coli* bacteraemia was more likely to be polyclonal in patients with severe immunocompromise given that humans often carry multiple *E. coli* strains simultaneously.^1^

We assembled a prospective cohort of immunocompetent and neutropaenic patients with *E. coli* bacteraemia. Whole genome sequencing (WGS) was performed on isolates and VF gene profiles, ST distribution, and isolate antibiogram data compared between patient groups.

## Methods

### Patients and study design

Adults admitted to University Hospital Southampton (UHS), UK, with *E. coli* bacteraemia were recruited prospectively within 2 weeks of the positive blood culture (BC) and allocated into two groups: (1) immunocompetent patients and; (2) neutropaenic patients (neutrophil count <1.0 × 10^9^/l within 24 h of BC sampling). Haematological malignancy, metastatic solid organ tumour/other immunocompromising conditions (e.g. inherent immunodeficiency syndromes or infection with human immunodeficiency virus), and immunosuppressant medications (oral/intra-venous steroids, disease modifying anti-rheumatic drugs, immunological therapies or chemotherapy) were exclusion criteria for admission to group 1. Patients who were discharged or deceased prior to screening were excluded. Charlson Comorbidity Index^11^ and severity of sepsis (severe inflammatory response syndrome scoring system)^12^ were calculated on admission. Presence of a urinary catheter and history of recurrent urinary tract infection (UTI) (defined as ≥2 episodes of UTI in last 6 months or ≥3 episodes of UTI in last 12 months),^13^ as well as date of discharge and in hospital death were recorded.

### Infection focus definitions

Infective foci were determined by the study physician following direct clinical consultation/review of laboratory and radiological data. Urinary tract infective foci (UTIF) were defined microbiologically (localised symptoms/signs with urinary *E. coli* culture – same antibiogram as bacteraemia isolate), radiologically (localised symptoms/signs with radiological findings suggesting UTIF), or clinically (localised symptoms/signs, microbiological/radiological investigations not performed or culture negative despite presence of urinary pyuria). In the neutropaenic group, ‘unknown infective focus’ was assigned when no clinical/radiological/microbiological evidence identified a focus. When performed, urine culture was *E. coli* culture negative in these patients.

### Bacterial strains and antimicrobial susceptibility testing

BCs were incubated (BacTAlert^®^ 3D microbial detection system, Biomerieux) and *E. coli* colonies identified by matrix-assisted laser desorption/ionisation time of flight (MALDI-TOF) mass spectrometry (Microflex, Bruker) following growth on cysteine lactose electrolyte deficient (CLED) agar (Oxoid). Antimicrobial susceptibilities were determined using Metascan Elite (MAST) with British Society for Antimicrobial Chemotherapy (BSAC) breakpoints.^14^ Isolates resistant to amoxicillin/piperacillin plus cefotaxime were screened for extended-spectrum beta-lactamase (ESBL) production utilising antimicrobial/inhibitor discs (Rosco).

Antimicrobial resistance scores comprised the number of antimicrobial agents to which the isolate was resistant. MDR was defined in line with international guidelines (non-susceptible to ≧1 agent in ≧3 antimicrobial categories).^15^

Urine microscopy (Sedimax platform, Menarini Diagnostics), culture and sensitivity testing (Metascan Elite) was performed. A urinary WCC >10/µl was considered elevated. Urinary isolates were confirmed as *E. coli* using MALDI-TOF mass spectrometry.

Bacteraemia and, where available, linked urinary isolates were sequenced.

### Determination of E. coli bacteraemia clonality

Random amplified polymorphic DNA (RAPD) fingerprinting was performed on isolates using a previously validated method.^16^ BC broths were sub-cultured onto CLED agar and incubated (5% CO2, 37 °C, 24 h). Following confirmation of *E. coli* growth, between 8 and 9 colonies per patient were randomly selected for RAPD. Two polymerase chain reactions (PCRs) were performed per colony (primers 1247^17^ [AAGAGCCCGT] and 1283^18^ [GCGATCCCCA]). Each 20 µl PCR reaction contained 1 µl of primer (final concentration 2 µM), 10 µl MyTaq Red Mix (Bioline) master mix, 6.5 µl PCR-grade water (Thermofisher) and 2.5 µl of DNA template (prepared by placing a 1 µl loop of colony into 50 µl of PCR-grade water and heating at 90 °C, 10 min). Cycling conditions for primers 1247 and 1283 were as follows: 95 °C for 10 min; 35 cycles of: 94 °C for 30 s, 38/36 °C for 30 s and 72 °C for 2 min; followed by 72 °C for 10 min (final elongation step). Amplification products were run on 0.7% agarose gels containing midori green (Geneflow) (90 V for 90 min) prior to image capture of PCR amplification products using a UV transilluminator linked to a digital camera.

### WGS and analyses

*E. coli* genomes were sequenced by Public Health England (PHE), Colindale (UK), using the Nextera sample preparation method with the standard 2× base sequencing protocol on a HiSeq instrument (IIllumina, San Diego, CA, USA), as described previously.^19^ This resulted in 2× paired-ended 100 bp length sequencing reads. SRST2 was used with standard parameters^20^ in conjunction with the VF (DoA: 05/08/2017)^21^ and *Escherichia coli* #1 multi-locus sequence typing (MLST)^22^ databases to determine VF gene profiles and STs, respectively. VF genes (31 in total) were included in the analysis if they were listed in the VF database^21^ and previously outlined as ExPEC-associated VFs in the literature.^1^, ^23^ Genomes were assembled and error-corrected using the A5 pipeline V20160825.^24^ Assembly metrics were generated using QUAST V4.6.3.^25^ Genome assemblies were annotated using Prokka V1.12^26^ using the – use_genus and a list of proteins derived from sequenced reference urinary pathogenic *E. coli* (UPEC) isolates with the – proteins flag. GFF annotations were used in conjunction with Prank^27^ as part of the Roary pipeline V3.8.0^28^ to generate core genome alignment. This utilised 1451 core genes out of a total 20,461 genes. The alignment was used in conjunction with FastTree V2^29^ and recompiled with duse_double to generate a maximum likelihood tree in .newick format using the gtr nt model. Phylogenetic tree visualisation and node editing was performed using Figtree V1.4.2.^30^ Paired sequencing reads utilised in the methods for this study are available from the Genome Sequence Archive (Preliminary accession: PRJCA001033). The data will become publicly available upon publication.

### Ethical considerations

The study was approved by the National Health Service Research Ethics Committee, North East – Tyne and Wear South (reference: 15/NE/0087) and the UHS Research and Development Department. Written informed consent was gained from patients prior to enrolment onto the study.

### Statistics

Parametrically- and non-parametrically-distributed continuous variables were summarised with mean+/− standard deviation (SD) or median (range/interquartile range), respectively. Unpaired Student's *t* test and Mann Whitney tests were used to compare parametrically and non-parametrically-distributed continuous data, respectively. Comparison of proportions across two groups was performed using Fisher's exact test. Chi squared (χ^2^) test for trend was used to compare proportions across three groups. In these analyses, no corrections were made for multiple comparisons.

Binomial logistic regression analysis was utilised to determine independent risk factors associated with UTIF vs. non-UTIF bacteraemia in immunocompetent subgroup analysis. Statistical analyses were performed in GraphPad Prism (version 7.0a) and SPSS (version 25.0).

## Results

### Study population and E. coli isolates

**Fig. 1:**
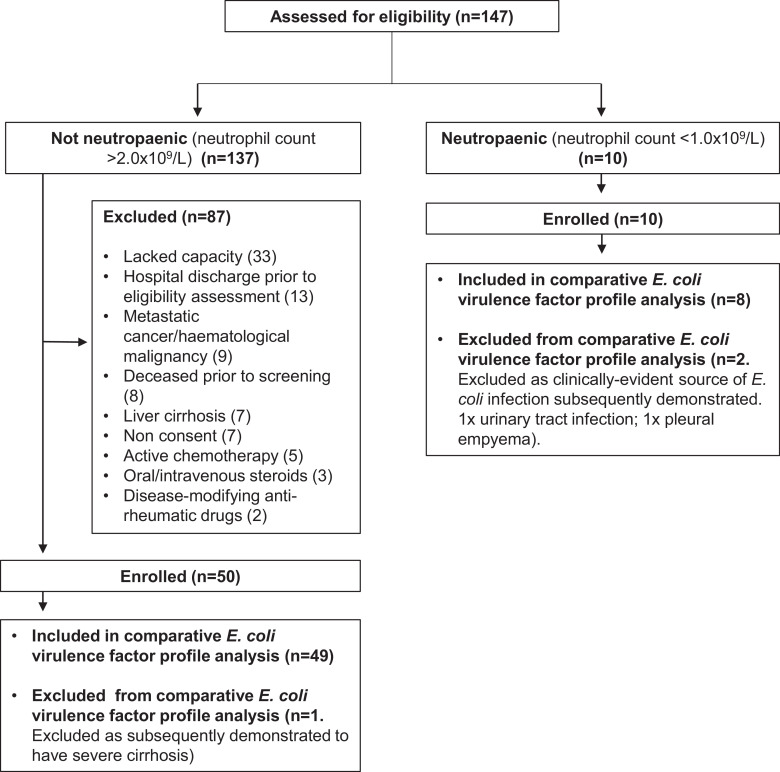
*E. coli* bacteraemia screening and recruitment chart.

147 consecutive patients with *E. coli* bacteraemia were screened between August 2015 and April 2016. 50 immunocompetent patients were enrolled representing 51 bacteraemia episodes (one patient had 2 bacteraemia episodes of different ST, separated by 46 days. Both isolates were included in inter-group VF gene comparison). 10 neutropaenic patients were enrolled representing 10 bacteraemia episodes (for causes of neutropaenia see Supplementary Table 1). Following withdrawals (Fig. 1), data from 49 immunocompetent (50 isolates) and 8 neutropaenic (8 isolates) patients were available for inter-group VF gene analysis.

**Table 1:** Baseline characteristics in patients with *E. coli* bacteraemia. Data available for all patients unless indicated*. Continuous variables expressed as mean +/− standard deviation or median with range. Proportions expressed as patient numbers with percentage in brackets. *P* values calculated with unpaired student's *t* test (a) or Mann Whitney test (b) for parametrically- and non-parametrically-distributed variables, respectively. *P* values for proportions calculated with Fisher's exact test. BP (blood presure); CKD (chronic kidney disease); COPD (chronic obstructive pulmonary disease); CVA (cerebrovascular event); INR (international normalised ratio); ITU (intensive care unit); MI (myocardial infarction); PVD (peripheral vascular disease); SIRS (systemic inflammatory response syndrome); TIA (transient ischaemic attack); UTI (urinary tract infection).

	Immunocompetent				*P* value	
Characteristic	1. All (*n* = 49)	2. Urinary focus (*n* = 23)	3. Non-urinary focus (*n* = 26)	4. Neutropaenic (unknown focus, *n* = 8)	1 × 4	2 × 3
Age (median, years)	70.1 (19.6–96.4)	70.1 (19.6–96.4)	69.3 (24.3–95.0)	63.5 (31.0–85.0)	0.54 (a)	0.93 (a)
Sex						
*Male*	19 (38.7)	6 (26.1)	13 (50)	5 (62.5)	0.26	0.14
*Female*	30 (61.2)	17 (73.9)	13 (50)	3 (37.5)		
Comorbidities						
*Charlson comorbidity index (mean)*	3.2 +/− 2.1	3.6 +/− 2.4	2.8 +/− 1.7	4.8 +/− 2.3	0.06 (a)	0.21 (a)
*Diabetes mellitus*	16 (32.7)	10 (43.5)	6 (23.1)	1 (12.5)	0.41	0.22
*Hypertension*	15 (30.6)	7 (30.4)	8 (31.8)	3 (37.5)	0.70	>0.99
*Previous CVA/TIA*	7 (14.3)	4 (17.4)	3 (11.5)	1 (12.5)	>0.99	0.69
*CHF*	4 (8.2)	3 (13.0)	1 (3.8)	0 (0)	>0.99	0.33
*Localised cancer*	4 (8.2)	2 (8.7)	2 (7.7)	1 (12.5)	0.54	>0.99
*Previous MI*	3 (6.1)	1 (4.3)	2 (7.7)	1 (12.5)	0.46	>0.99
*CKD*	2 (4.1)	2 (8.7)	0 (0)	0 (0)	>0.99	0.22
*COPD*	2 (4.1)	2 (8.7)	0 (0)	0 (0)	>0.99	0.22
*PVD*	0 (0)	0 (0)	0 (0)	0 (0)	>0.99	>0.99
*Dementia*	0 (0)	0 (0)	0 (0)	0 (0)	>0.99	>0.99
*Hemi-plegia*	0 (0)	0 (0)	0 (0)	0 (0)	>0.99	>0.99
*Connective tissue disorder*	0 (0)	0 (0)	0 (0)	0 (0)	>0.99	>0.99
*Peptic ulcer disease*	0 (0)	0 (0)	0 (0)	0 (0)	>0.99	>0.99
*Urinary catheter*	4 (8.2)	3 (13.0)	1 (3.8)	0 (0)	>0.99	0.33
*History of recurrent UTIs*	6 (12.2)	6 (26.1)	0 (0)	0 (0)	0.58	0.01
*Antimicrobials in last 28 days*	18/48 (37.5)	11 (47.8)	7/25 (28.0)	5 (62.5)	0.25	0.23
Admission sepsis criteria						
*Patients meeting SIRS sepsis definition*	44 (89.8)	21 (91.3)	23 (88.5)	8 (100)	>0.99	>0.99
*Temperature (mean,°C)*	38.5 +/− 1.0 (*n* = 48)	38.5 +/− 1.1	38.4 +/− 1.1	38.6 +/− 0.9	0.75 (a)	0.83 (a)
*Heart rate (mean, beats per minute)*	107.7 +/− 19.4	107.9 +/− 21.1	107.5 +/−18.2	113.3 +/− 22.5	0.47 (a)	0.94 (a)
*Respiratory rate (mean, breaths per minute)*	24.7 +/− 7.2 (*n* = 48)	23.0 +/− 6.2	26.2 +/− 7.8	22.4 +/− 5.9	0.36 (b)	0.14 (b)
*White cell count (mean, ×10^9^/l)*	12.8 +/− 6.2	13.4 +/− 5.8	12.3 +/− 6.7	0.13 +/− 0.21	<0.0001 (a)	0.53 (a)
*Acutely altered mental status*	8 (16.3)	6 (26.1)	2 (8.3)	1 (12.5)	>0.99	0.13
*Severe sepsis*	32 (65.3)	11 (47.8)	21 (80.8)	8 (100)	0.09	0.02
*Creatinine >*176.8µmol/l	14 (28.6)	8 (34.8)	6 (23.1)	1 (12.5)	0.67	0.53
*Lactate* >2 mmol/l	17/41 (41.5)	6/21 (28.6)	11/20 (55)	Not available	–	0.12
*Platelet count* <100 × 10^9^/l	2/48 (4.2)	1 (4.3)	1/25 (4)	8 (100)	<0.0001	>0.99
*INR >1.5*	13/37 (35.1)	2/17 (11.8)	11/20 (55)	Not available	–	0.01
*Bilirubin* >68.42µ mol/l	5/47 (10.6)	0/22 (0)	5/25 (20)	1 (12.5)	>0.99	0.05
*Systolic BP* <90 mmH g *or reduction in systolic BP* >40 mmH g *from baseline*	18/48 (37.5)	6 (26.1)	12/25 (48)	2/7 (28.5)	>0.99	0.14
*Septic shock*	8 (16.3)	3 (13)	5 (19.2)	1 (12.5)	>0.99	0.71
*ITU admission*	8 (16.3)	3 (15)	5 (19.2)	1 (12.5)	>0.99	0.71
Length of stay (median, days)	7 (3–84)	7 (3–78)	9 (3–84)	24 (12–147)	<0.01	0.31
In hospital death	2 (4.1)	1 (4.3)	1 (3.8)	0 (0)	>0.99	>0.99

Foci of *E. coli* bacteraemia included UTIF (*n* = 23; 70%, 17% and 13% proven microbiologically, radiologically and clinically, respectively), non-UTIF (*n* = 26) and neutropaenic patients with unknown focus of infection (NPUFI) (*n* = 8). Analysis of WGS data demonstrated that 15/16 linked urinary isolates from patients with microbiologically-proven UTI shared the same ST as the bacteraemia strain. Baseline characteristics, admission sepsis severity parameters, and mortality/length of stay data, are outlined in Table 1. Significantly more immunocompetent patients with UTIF vs. non-UTIF had a history of recurrent UTI, while patients with non-UTIF vs. UTIF were more likely to have severe sepsis on admission (because of hyperbilirubinaemia and coagulopathy in patients with cholangitis/cholecystitis). WCC and platelet counts were significantly lower in neutropaenic patients as expected.

### E *coli* bacteraemia is monoclonal in neutropaenic and non-neutropaenic patients

RAPD analysis was performed on 8–9 *E. coli* colonies (growing on CLED agar) for 14/23, 20/26 and 8/8 bacteraemia isolates from patients with UTIF, non-UTIF, and NPUFI, respectively (representative example for isolate 43 demonstrated in Supplementary Fig. 1). For all patients, intra-patient *E. coli* colonies differed by ≤1 band across the 2 RAPD primers utilised, consistent with a low probability of genomic differences (when compared to WGS) as previously described.^16^ The possibility of polyclonal *E. coli* bacteraemia was thus excluded prior to selection of a single colony per patient for WGS.

### Bacteraemia isolates originating from the urinary tract have distinct VF gene profiles compared with isolates from non-urinary foci

**Fig. 2:**
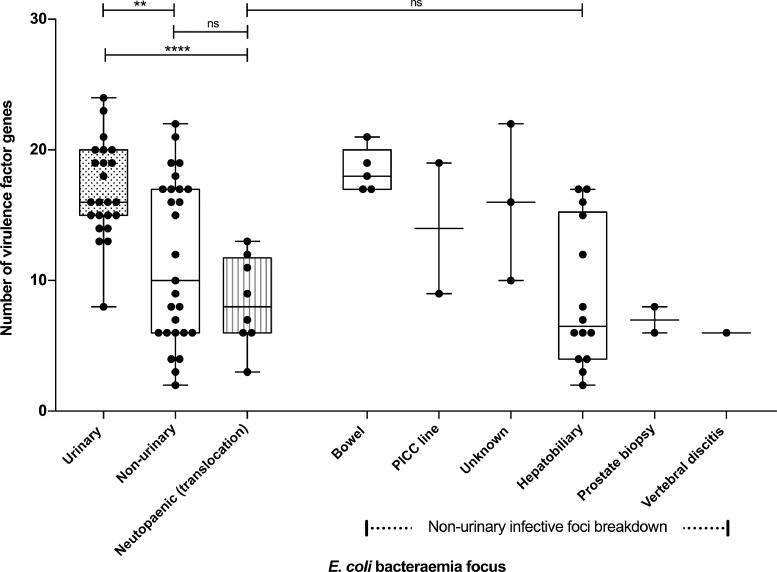
Number of virulence factor genes amongst *E. coli* isolates from immunocompetent and neutropaenic patients according to infective focus. Box and whisker plots indicate number of virulence factor genes amongst isolates derived from specific infective foci. Isolates derived from non-urinary foci subdivided further into sub-groups as indicated. Number of virulence factor genes between groups compared with Mann–Whitney test (**p < 0.01; ****p < 0.0001; ns – non-significant). PICC (peripherally-inserted central catheter).

Univariate analysis demonstrated that the median number of VF genes was significantly higher amongst isolates from immunocompetent patients (*n* = 50, all infective foci) compared to NPUFI (*n* = 8) (median number of VF genes 15.5, range 2–24, and 8, 3–13, respectively, *p* = 0.0076). Within the immunocompetent group, the median number of VF genes was significantly higher amongst isolates derived from UTIF (*n* = 23) vs. non-UTIF (*n* = 27) (16, range 8–24, and 10, 2–22, respectively, *p* = 0.0058). Isolates originating from NPUFI had a significantly lower median number of VF genes (8, 3–13) compared with isolates from immunocompetent patients originating from UTIF (16, 8–24, *p* < 0.0001) (Fig. 2).

Binomial logistic regression analysis demonstrated that number of VF genes (OR 1.21, 95% CIs 1.01–1.46, *p* = 0.039) and recurrent UTI history/presence of urinary catheter (OR 12.82, 95% CIs 1.24–132.65, *p* = 0.032) were independent predictors of bacteraemia originating from UTIF in a model inclusive of number of VF genes present within the *E. coli* isolate, and host variables associated with susceptibility to bacteraemia and UTI including gender, age (years), Charlson Comorbidity Index, history of recent antimicrobials (28 days prior to bacteraemia), and recurrent UTI history/presence of a urinary catheter (Table 2) 31, 32, 33. For every unit increase in the VF gene number, the odds of a bacteraemia isolate being derived from a urinary focus increased by 1.21 times.

**Table 2:** Binomial logistic regression analysis for predictors of *E. coli* bacteraemia arising from urinary vs. non-urinary foci in immunocompetent patients. Total number of observations used in analysis, *n* = 50. UTI (urinary tract infection).

Variable	*B*	S.E.	Odds ratio	Wald *p* value	95% CIs
Number of virulence factors	0.193	0.093	1.21	0.039	1.01–1.46
Age (years)	−0.014	0.042	0.99	0.735	0.91–1.07
Charlson Comorbidity Index	0.466	0.357	1.49	0.281	0.79–3.21
Antimicrobials in last 28 days	1.622	0.879	5.06	0.065	0.91–28.35
Recurrent UTI history or urinary catheter	2.551	1.192	12.82	0.032	1.24–132.65
Female gender	0.797	0.773	2.22	0.303	0.49–10.10
Constant	−4.722	2.881	0.01	0.101	

*x*^2^ 23.23, df = 6, *p* = 0.001. Nagelkerke R2 49.7%. Hosmer and Lemeshow test *p* = 0.336 Classification accuracy 78%.

Univariate analysis demonstrated that the prevalence of *papA, papC, papE/F, papG, agn43, tia, iut, fyuA, kpsM* and *sat* genes was significantly higher amongst isolates originating from UTIF vs. non-UTIF, while prevalence of *papC, papE/F, papG, agn43, tia, fyuA, hlyA, usp* and *clb* was significantly higher amongst isolates originating from UTIF vs. NPUFI (Table 3). Full VF gene profiles for each isolate are outlined in Supplementary Table 2.

**Table 3:** Distribution of virulence factor genes amongst 58 *E. coli* isolates from immunocompetent (urinary vs. non-urinary infective focus) and neutropaenic (unknown focus) patient groups. Proportions expressed as number of isolates with virulence factor gene detected (percentage in brackets). Proportions across groups compared with Chi-squared test for trend (a) and Fisher's exact test (b) as indicated. na (not applicable).

Virulence factor gene	Immunocompetent			*P* value			
	1. Urinary (*n* = 23)	2. Non-urinary (*n* = 27)	3. Neutropaenic (unknown focus, *n* = 8)	1–3^a^	1 × 2^b^	1 × 3^b^	2 × 3^b^
Adhesins							
*afa/draBC* (afimbrial adhesins)	0 (0)	1 (4)	1 (13)	0.11	>0.99	0.26	0.42
*ecpA* (*E. coli* common pilus)	23 (100)	24 (89)	7 (88)	0.12	0.24	0.26	>0.99
*focA* (F1C fimbriae)	3 (13)	3 (11)	0 (0)	0.36	>0.99	0.55	>0.99
P fimbriae genes							
*papA*	18 (78)	9 (33)	3 (38)	<0.01	<0.01	0.07	>0.99
*papC*	22 (96)	12 (44)	1 (13)	<0.0001	<0.001	<0.0001	0.21
*pap E/F*	22 (96)	13 (48)	1 (13)	<0.0001	<0.001	<0.0001	0.11
*papG*	22 (96)	11 (41)	1 (13)	<0.0001	<0.0001	<0.0001	0.22
*sfaA* (S fimbriae)	1 (4)	2 (7)	0 (0)	0.62	>0.99	>0.99	>0.99
*tsh* (temperature sensitive hamagglutinin)	0 (0)	1 (4)	1 (13)	0.11	>0.99	0.26	0.42
*fimH* (type 1 fimbriae)	23 (100)	24 (89)	7 (88)	0.12	0.24	0.26	>0.99
*agn43* (antigen 43)	20 (87)	13 (48)	3 (38)	<0.01	<0.01	0.01	>0.99
*tia* (hek/tia adhesin and invasin)	18 (78)	13 (48)	2 (25)	<0.01	0.04	0.01	0.42
Iron-acquisition systems							
*iutA* (aerobactin receptor)	19 (83)	10 (37)	4 (50)	0.01	<0.01	0.15	0.69
*sitA* (peri-plasmic iron binding protein)	22 (96)	22 (81)	6 (75)	0.09	0.19	0.16	>0.99
*iroN* (salmochellin receptor)	12 (52)	11 (41)	2 (25)	0.15	0.40	0.24	0.68
*ireA* (siderophore receptor)	5 (22)	4 (15)	0 (0)	0.17	0.72	0.29	0.55
*fyuA* (yersiniabactin receptor)	23 (100)	20 (74)	5 (63)	<0.01	0.01	0.01	0.67
Protectins and Invasins							
*kpsM* (group 2 capsule)	22 (96)	17 (63)	6 (75)	0.04	<0.01	0.16	0.68
*ompA* (outer membrane protein A)	23 (100)	27 (100)	8 (100)	na	>0.99	>0.99	>0.99
*ibeA* (invasion of brain endothelium A)	1 (4)	4 (15)	0 (0)	0.83	0.35	>0.99	0.55
*tcpc* (toll receptor inhibitor)	9 (39)	6 (22)	0 (0)	0.02	0.20	0.07	0.31
Toxins							
*cdtB* (cytolethal distending toxin)	2 (9)	4 (15)	1 (13)	0.62	0.67	>0.99	>0.99
*cnf1* (cytotoxic necrotising factor)	8 (35)	7 (26)	0 (0)	0.08	0.76	0.08	0.16
*astA* (heat stable enterotoxin 1)	3 (13)	1 (4)	0 (0)	0.14	0.33	0.55	>0.99
*hlyA* (haemolysin A)	12 (52)	7 (26)	0 (0)	<0.01	0.09	0.01	0.16
*sat* (secreted autotrasporter toxin)	15 (65)	7 (26)	4 (50)	0.11	0.01	0.68	0.39
*usp* (uropathogen-specific protein)	17 (74)	13 (48)	2 (25)	<0.01	0.08	0.03	0.42
*clb* (colibactin synthesis)	9 (39)	8 (30)	0 (0)	<0.01	0.20	0.02	0.16
*pic* (serine protease)	4 (17)	5 (19)	0 (0)	0.39	>0.99	0.55	0.31
*vat* (vacuolating toxin)	10 (43)	11 (41)	2 (25)	0.38	0.78	0.43	0.68
Others							
*fliC* (flagellin variant)	1 (4)	1 (4)	0 (0)	0.62	>0.99	>0.99	>0.99

Strains belonging to MLST STs 12 and 69 were more frequent in immunocompetent bacteraemia originating from UTIF vs. non-UTIF (17.4% vs 0%, *p* = 0.04, and 21.7% vs. 0%, *p* = 0.02, respectively. See Table 4). Antimicrobial resistance scores and the proportion of MDR isolates were not significantly different between isolates from UTIF and non-UTIF in immunocompetent patients. Ciprofloxacin resistance was significantly more prevalent in NPUFI vs. isolates from immunocompetent patients with UTIF (75% vs 21.7%, *p* = 0.012) (Table 4), reflecting the use of ciprofloxacin prophylaxis in patients with haematological malignancy.

**Table 4:** Distribution of *E. coli* virulence factor genes (including subgroups), common STs, and antimicrobial resistance among isolates from clinical groups. Proportions compared using Fisher's exact test. ESBL (extended-spectrum beta-lactamase); ExPEC (extra-intestinal pathogenic *E. coli*); ST (sequence type); MDR (multi-drug-resistant). Isolates classified as ‘intermediate’ on phenotypical sensitivity testing were considered resistant for this analysis. MDR defined in line with international consensus guidelines, i.e. non-susceptible to ≧1 agent in ≧3 antimicrobial categories including aminoglycosides, anti-MRSA cephalosporins, anti-pseudomonal penicillins with beta-lactamase inhibitors, carbapenems, non-extended spectrum cephalosporins (i.e. 1st and 2nd generation), extended-spectrum cephalosporins (i.e. 3rd and 4th generation), cephamycins, fluoroquinolones, trimethroprim-sulphamethoxazole, glycyclines, monobactams, penicillins, penicillins with betalactamase inhibitors, chloramphenicol, phosphonic acids and colistin.

	1. Immunocompetent				*P* value			
Characteristic	1a. All (*n* = 50)	1b. Urinary focus (*n* = 23)	1c. Non-urinary focus (*n* = 27)	2. Neutropaenic (*n* = 8)	1a × 2	1b × 1c	1b × 2	1c × 2
VF gene number, median (range)								
*Total VF genes*	15.5 (2–24)	16 (8–24)	10 (2–22)	8 (3–13)	0.01	0.01	<0.0001	0.28
*Adhesin VF genes*	7 (1–9)	8 (3–9)	4 (1–9)	3 (1–7)	0.01	<0.001	<0.0001	0.37
*Iron-acquisition VF genes*	3 (0–5)	3 (2–5)	3 (0–5)	3 (1–3)	0.18	<0.01	0.02	0.73
*Protectin/invasin VF genes*	2 (1–3)	2 (1–3)	2 (1–3)	2 (1–2)	0.09	0.08	0.01	0.50
*Toxins VF genes*	3 (0–7)	3 (1–7)	2 (0–6)	1 (0–2)	0.02	0.05	0.001	0.21
Distribution of most frequent STs, *n* (%)								
*ST 73*	6 (12)	3 (13)	3 (11)	0 (0)	0.58	>0.99	0.55	>0.99
*ST 12*	4 (8)	4 (17)	0 (0)	0 (0)	>0.99	0.04	0.55	>0.99
*ST 127*	5 (10)	2 (9)	3 (11)	0 (0)	>0.99	>0.99	>0.99	>0.99
*ST 131*	13 (26)	6 (26)	5 (19)	2 (25)	>0.99	0.73	>0.99	0.65
*ST 69*	5 (10)	5 (22)	0 (0)	0 (0)	>0.99	0.02	0.29	>0.99
*ST648*	3 (6)	0 (0)	3 (11)	0 (0)	>0.99	0.24	>0.99	>0.99
Antimicrobial resistance score, median (range)	1 (0–11.5)	2 (0–11.5)	1 (0–10.5)	4 (0–8)	0.28	0.32	0.49	0.20
Meet MDR definition, *n* (%)	15 (30)	8 (35)	7 (26)	4 (50)	0.42	0.55	0.68	0.23
Resistant to, *n* (%):								
*Amoxicillin*	27 (54)	14 (61)	13 (48)	5 (63)	0.72	0.41	>0.99	0.69
*Piperacillin*	21 (42)	12 (52)	9 (30)	5 (63)	0.45	0.25	0.70	0.22
*Co-amoxiclav*	10 (20)	6 (26)	4 (15)	2 (25)	0.66	0.48	>0.99	0.60
*Piperacillin-tazobactam*	5 (10)	3 (13)	2 (7)	2 (25)	0.25	0.65	0.58	0.22
*Cefuroxime*	10 (20)	5 (21)	5 (19)	2 (25)	0.65	>0.99	>0.99	0.65
*Cefotaxime*	8 (16)	4 (17)	4 (15)	0 (0)	0.58	>0.99	0.55	0.55
*Ceftazidime*	6 (12)	4 (17)	2 (7)	0 (0)	0.58	0.39	0.55	>0.99
*Ciprofloxacin*	10 (20)	5 (22)	5 (19)	6 (75)	<0.01	>0.99	0.01	<0.01
*Gentamicin*	6 (12)	4 (17)	2 (7)	2 (25)	0.30	0.39	0.63	0.22
*Meropenem*	0 (0)	0 (0)	0 (0)	0 (0)	>0.99	>0.99	>0.99	>0.99
*Ertapenem*	0 (0)	0 (0)	0 (0)	0 (0)	>0.99	>0.99	>0.99	>0.99
*Chloramphenicol*	9 (18)	4 (17)	5 (9)	2 (25)	0.64	>0.99	0.63	0.65
*Trimethroprim-sulfamethoxazole*	13 (26)	7 (30)	6 (20)	4 (50)	0.22	0.54	0.41	0.19
*Colistin*	0 (0)	0 (0)	0 (0)	0 (0)	>0.99	>0.99	>0.99	>0.99
*Temocillin*	4 (8)	2 (9)	2 (7)	1 (13)	0.54	>0.99	>0.99	0.55
*n* (%) meeting ExPEC definition	38 (76)	23 (100)	15 (56)	3 (38)	0.04	<0.001	<0.001	0.44
Phenotypical +/− Genotypical detection of ESBL, *n* (%)	7 (14)	4 (17)	3 (11)	0 (0)	0.18	0.69	0.55	>0.99

### Bacteraemia isolates originating from NPUFI are similar to those originating from non-urinary foci in immunocompetent patients

Bacteraemia isolates from immunocompetent patients originating from non-UTIF had similar numbers of VF genes to those from NPUFI (median number of VF genes 10, range 2–22, and 8, 3–13, respectively, *p* = 0.28). In addition, no significant differences in the prevalence of individual VF genes (Table 3), groups of VF genes, or in distribution of common STs were observed between these groups (Table 4). The proportion of non-UTIF- and NPUFI-derived bacteraemia isolates meeting the previously-defined ExPEC definition [5] was 56% and 38%, respectively (*p* = 0.44) demonstrating that strains that did not meet the ExPEC definition were responsible for a large proportion of disease amongst these patient groups. By comparison, 100% of bacteraemia isolates derived from UTIF met the ExPEC definition (*n* = 23/23 UTIF vs *n* = 15/27 non-UTIF, *p* < 0.001; *n* = 23/23 UTIF vs *n* = 3/8 NPUFI, *p* < 0.001) (Table 4).

Total antimicrobial resistance scores and the proportion of MDR isolates were not significantly higher in NPUFI-associated isolates compared with non-UTIF isolates from immunocompetent patients. Ciprofloxacin resistance was significantly more frequent amongst NPUFI-associated isolates compared with non-UTIF isolates from immunocompetent patients (75% vs.18.5%, *p* = 0.006) (Table 4).

### Associations between VF genes, antimicrobial resistance and STs

**Fig. 3:**
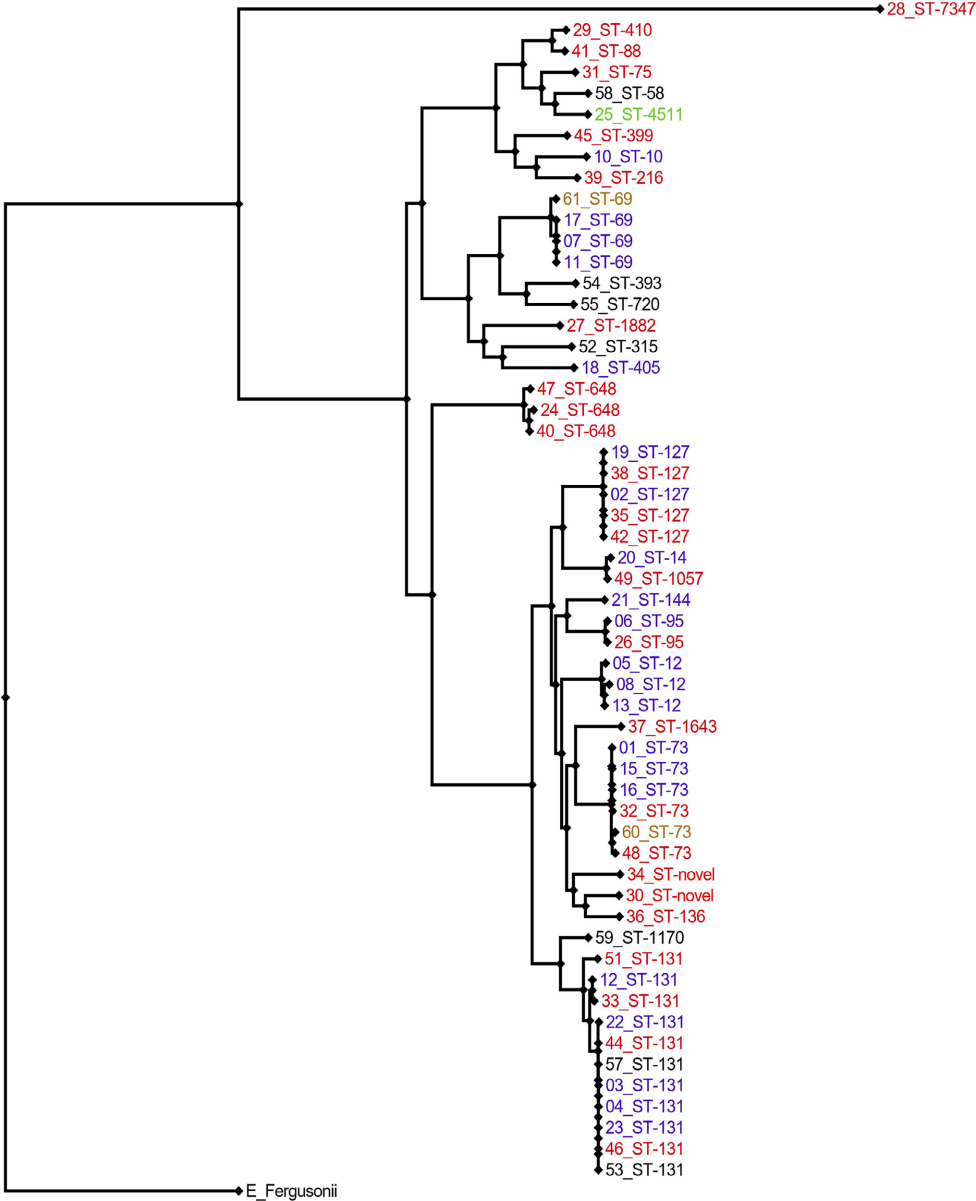
Core-genome maximum-likelihood phylogenetic tree of *E. coli* bacteraemia isolates. Tree constructed with the generalised time-reversible model using FastTree V2.1 and features 56/61 isolates. Isolate numbers and associated sequence type (ST) data are presented. Bacteraemia isolates associated with urinary tract foci, non-urinary tract foci (immunocompetent patients) and unknown foci (neutropaenic patients) are indicated in purple (01–23), red (24–51) and black (52–59), respectively. Isolates excluded from the inter-patient VF gene analysis are indicated in green (25 – immunocompetent patient with cirrhosis) and gold (60–61 – neutropaenic patients with demonstrable focus of infection). Novel STs indicate the emergence of a new sequence type (to be classified) due to unambiguous, multi-locus ST-allelic variation. Reads from Isolates 9,14,43,50 and 56 were unable to be resolved into draft genome assemblies using the A5 pipeline and were excluded from phylogenetic inference. (For interpretation of the references to colour in this figure legend, the reader is referred to the web version of this article.)

30 STs were identified (analysis included isolates from patients withdrawn for the purposes of inter-patient VF gene analysis, *n* = 61), the most frequently occurring being ST131 (*n* = 13), ST73 (*n* = 6), ST69 (*n* = 5), ST127 (*n* = 5), and ST12 (*n* = 4). Two novel STs were identified (Supplementary Fig. 2). A phylogenetic tree was constructed for bacteraemia isolates (Fig. 3).

**Fig. 4:**
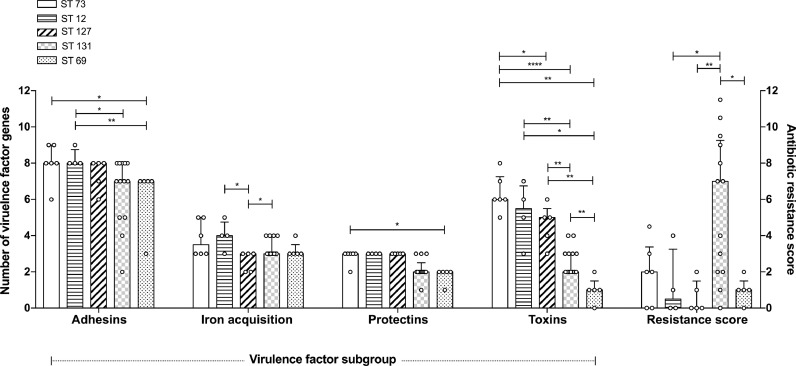
Virulence factor gene subgroups and antimicrobial resistance scores amongst most prevalent *E. coli* sequence types (STs). Bars represent median values with interquartile range (error bars). Distribution of virulence factor numbers between STs compared with Mann–Whitney test. Significant results indicated (*p < 0.05; **p < 0.01; ***p < 0.001; ****p < 0.0001).

Total median number of VF genes of isolates within STs 12, 73, 127, 131 and 69 were: 20 (range 19–23), 20.5 (19–23), 17 (16–20), 15 (10–18) and 13 (7–14), respectively. Significant differences in numbers of VF genes (Supplementary Fig. 2) and subgroups of VF genes (particularly pronounced across adhesin and toxin categories – Fig. 4) were evident on comparing isolates belonging to certain STs. Median antimicrobial resistance scores were highest amongst isolates belonging to ST131 (7, range 0–11.5). Antimicrobial resistance scores did not correlate with number of VF genes across STs (Fig. 4). Of the isolates, 7/61 (11.5%) carried ESBL enzymes (6/7 ST131 and 1/7 ST648) and 19/61 (31.1%) were MDR (Table 4 and Supplementary Table 3).

## Discussion

In our study, bacteraemia-associated *E. coli* strains originating from UTIF harboured significantly more VF genes than non-UTIF- and NPUFI-associated strains. Number of VF genes was an independent predictor of bacteraemia derived from UTIF in immunocompetent patients with the odds of bacteraemia secondary to UTIF increasing by 1.21 times for every unit increase in VF gene number. A broad range of STs were identified with STs 12, 69, 73, 127 and 131 accounting for 51% of isolates, a finding that is in keeping with recently published UK data.^8^

VFs associated with UTI-associated *E. coli* strains are well described^23^^,^^34^, ^35^, ^36^ but analyses comparing VF gene profiles of bacteraemia strains originating from well-defined infective foci are rare. Like us, Micenková et al. found more VF genes amongst UTIF- compared with non-UTIF bacteraemia isolates.^37^ In our study, univariate analysis of VF genes demonstrated that UTIF-associated isolates more frequently harboured *papA, papC, papE/F, papG* (P fimbriae), *agn43* and *tia* (adhesins), *iutA, fyuA* (iron-acquisition-related genes), *kpsM* (capsule) and the *sat* toxin compared to non-UTIF isolates, and more frequently harboured *papC, papE/F, papG, agn43, tia, fyuA, hlyA* (haemolysin A), *usp* (uropathogen-specific protein) and *clb* (colibactin synthesis gene) compared to NPUFI-associated isolates. These findings strengthen previously described associations between P fimbriae-encoding genes and uro-epithelial adhesion/associations with cystitis or pyelonephritis-causing strains,^36^, ^38^ *iutA*/*hlyA* and pyelonephritis-causing strains,^34^ and *kpsM* (capsule)/P fimbriae and their relationships with UTI-associated bacteraemia.^32^ Although previously associated with UTI/pyelonephritis,^36^ *afa/draBC* (Dr-binding adhesins), *ibeA* (invasion of brain endothelium) and *sfaA* (S fimbriae) were not more prevalent amongst UTIF-associated isolates in our study. Our data strengthen previous observations relating to UTIF-specific VF genes but also reveal that, in UTIF-associated bacteraemia, *agn43, tia, fyuA* and *usp* may be of significance.

Isolates from immunocompetent patients originating from non-UTIF were not significantly dissimilar to isolates from NPUFI in relation to total number of VF genes or distribution of individual VF genes. Only 56% and 38% of isolates from immunocompetent patients with non-UTIF and NPUFI, respectively, met the utilised genomic definition for ExPEC^5^ compared to 100% of isolates from urinary foci. These data demonstrate the broad diversity of strains associated with invasive disease outside of the context of UTI and support the hypothesis that non-UTIF and NPUFI-derived isolates likely originated from the same location, i.e. the gastro-intestinal tract.

The number of VF genes amongst isolates from NPUFI were low and 11/31 VF genes (*focA, sfaA, ireA, ibeA, tcpc, cnf1, astA, hlyA, clb, pic* and *fliC*) were completely absent. Recently published data comparing the VF gene profiles of bowel translocation-associated bacteraemia isolates to faecal controls in patients with haematological malignancy demonstrated that specific clusters of VF genes may be associated with increased translocation potential.^39^ In our study which focused specifically on bacteraemia-associated strains, no individual VF genes were more frequent amongst isolates derived from NPUFI compared with UTIF or non-UTIF. Put together, these findings suggest that although bowel translocation-associated isolates derived from immunocompromised patients may possess specific VFs that enable this process, these isolates generally harbour fewer ExPEC-associated VF genes compared with bacteraemia-associated isolates derived from immunocompetent hosts.

It seems likely that translocation events occur secondary to damage to the structural integrity of the intestinal mucosa or as a result of compromised mucosal or systemic immunity, or both.^9^, ^36^, ^38^, ^39^, ^40^ Interestingly, a large proportion of isolates associated with bacteraemia secondary to non-UTIF in immunocompetent patients were caused by isolates with low numbers of VF genes. The majority of these isolates were associated with intra-abdominal pathologies where the physical integrity of viscera and associated structures is often compromised due to the underlying pathology, e.g. severe inflammation +/− mechanical obstruction in cholecystitis/cholangitis. Under these circumstances *E. coli* isolates with low numbers of VFs may be able to translocate easily into the vascular system.^41^

Key strengths of this study include its prospective design, the distinction between immunocompetent/neutropaenic groups, the rigorous methods utilised to assign infective foci, the use of logistic regression, and the application of WGS to determine VF gene profiles. The small NPUFI group (a group that was difficult to recruit) was the main limitation and likely reduced the power to detect differences in VF gene distribution between isolates derived from neutropaenic and immunocompetent sub-groups. Additionally, the mode of infecting strain acquisition was not determined and thus a comparative analysis of community vs. nosocomially-acquired strains was not possible in this study.

In conclusion, *E. coli* bacteraemia strains associated with UTIF have enriched VF gene profiles compared to those from non-UTIF and NPUFI. Strains are genomically diverse and in this study non-UTIF-associated bacteraemia in immunocompetent patients was frequently caused by strains that did not meet the utilised genomic definition for ExPEC. Mapping the diversity of bacteraemia-causing strains will inform targeted or universal preventative strategies. Future vaccine development will depend upon these data to ensure adequate coverage of strains associated with site-specific disease.

## Funding

This work was supported by a University of Southampton Research Management Committee Award, with additional funding provided by the Department of Health, UK. The National Institute for Health Research (NIHR), UK, funded the salary of APD throughout the duration of the research programme (NIHR Academic Clinical Fellowship Scheme). APD is a Wellcome Trust Research Training Fellow (grant number 203581/Z/16/Z), and RCR is a NIHR Senior Investigator (grant number NF-SI-0617-10010).

## Conflicts of interest

APD, AKP, RJH, KB, JRL, KCS and RCR: No reported conflicts.

## Appendix Supplementary materials

Image, application 1
