# Supplementary material for: Genomes of Escherichia coli bacteraemia isolates originating from urinary tract foci contain more virulence-associated genes than those from non-urinary foci and neutropaenic hosts
Source: J Infect. 2018 Dec;77(6):534–43. doi: 10.1016/j.jinf.2018.10.011 (PMC6293314; doi:10.1016/j.jinf.2018.10.011)
Supplement: Supplementary file 1 [file mmc1.docx]

**Supplementary Figure 1. Random amplified polymorphic DNA (RAPD).** Representative example of RAPD (*E. coli* isolate 43) typing of 9 *E. coli* colonies chosen at random from CLED agar plate from which blood culture broth was directly inoculated and streaked. M – 1kb molecular weight ladder; P – positive control (*E. coli* strain, different donor); N - negative control; - empty lane. Primer 1 (1247 - AAGAGCCCGT); Primer 2 (1283 - GCGATCCCCA).

**Supplementary Figure 2. Number of *E. coli* virulence factor genes across multilocus sequence type (MLST) groups.** Box and whisker plots of virulence factor gene numbers of isolates belonging to different MLST groups. Median values compared using Mann-Whitney test and significant results demonstrated (**p*<0.05; ***p*<0.01; ****p*<0.001).

| ***E. coli* isolate** | **Bacteraemia source** | **Cause of neutropaenia** | **Neutrophil count x10**^9^**/L (result within 24 hour of BC)** | **Duration of neutropaenia prior to positive BC (days)** |
| --- | --- | --- | --- | --- |
| 52 | N-T | Chemotherapy for AML | 0.0 | 62 |
| 53 | N-T | Chemotherapy prior to auto-graft BMT for myeloma | 0.0 | 3 |
| 54 | N-T | Chemotherapy for AML | 0.0 | 87 |
| 55 | N-T | Chemotherapy for T-cell lymphoma | 0.5 | 0 |
| 56 | N-T | AML and hydroxycarbamide | 0.0 | 30 |
| 57 | N-T | Chemotherapy prior to auto-graft BMT for follicular lymphoma | 0.1 | 1 |
| 58 | N-T | Chemotherapy prior to auto-graft BMT for multiple myeloma | 0.0 | 2 |
| 59 | N-T | Chemotherapy prior to allo-graft BMT for CML | 0.0 | 9 |
| 60 | N-K | Chemotherapy for stage 4 ovarian cancer | 0.9 | 0 |
| 61 | N-K | Chemotherapy for diffuse large B-cell lymphoma | 0.1 | 0 |

**Supplementary Table 1. Causes of *E. coli* bacteraemia in neutropaenic study patients.** Focus of infection resulting in bacteraemia: N-T - neutropaenic, unknown focus (translocation); N-K - neutropaenic, known focus and excluded from comparative VF analysis. AML (acute myeloid leukaemia); BC (blood culture) BMT (bone marrow transplant); CML (chronic myeloid leukaemia).

| ***E. coli* isolate** | **Bacteraemia source** | **MLST ST** | **Adhesins** | | | | | | | | | | | | **Iron**  **Acquisition** | | | | | **Protectins &**  **Invasins** | | | | **Toxins** | | | | | | | | | **Other** | **Number of VF genes** |
| --- | --- | --- | --- | --- | --- | --- | --- | --- | --- | --- | --- | --- | --- | --- | --- | --- | --- | --- | --- | --- | --- | --- | --- | --- | --- | --- | --- | --- | --- | --- | --- | --- | --- | --- |
|  |  |  | ***fimH*** | ***ecpA*** | ***agn43*** | ***papE/F*** | ***papG*** | ***papC*** | ***tia*** | ***papA*** | ***focA*** | ***sfaA*** | ***tsh*** | ***afa/draBC*** | ***sitA*** | ***fyuA*** | ***iutA*** | ***iroN*** | ***ireA*** | ***ompA*** | ***kpsM*** | ***tcpc*** | ***ibeA*** | ***usp*** | ***sat*** | ***vat*** | ***hlyA*** | ***clb*** | ***cnf1*** | ***pic*** | ***cdtB*** | ***astA*** | ***fliC*** |  |
|  |  |  |  |  |  |  |  |  |  |  |  |  |  |  |  |  |  |  |  |  |  |  |  |  |  |  |  |  |  |  |  |  |  |  |
| 1 | U | 12 | + | + | + | + | + | + | + | + | + | - | - | - | + | + | + | + | - | + | + | + | - | + | + | + | + | + | + | + | - | - | - | 23 |
| 2 | U | 127 | + | + | - | + | + | + | + | - | - | - | - | - | + | + | - | - | - | + | + | + | - | + | - | + | + | + | + | - | - | - | - | 16 |
| 3 | U | 131 | + | + | - | + | + | + | + | + | - | - | - | - | + | + | + | + | - | + | + | - | - | + | + | - | - | - | - | - | - | - | - | 15 |
| 4 | U | 131 | + | + | - | + | + | + | + | + | - | - | - | - | + | + | + | + | - | + | + | - | - | + | + | - | - | - | - | - | - | - | - | 15 |
| 5 | U | 12 | + | + | + | + | + | + | + | + | - | - | - | - | + | + | - | + | - | + | + | + | - | + | - | + | + | + | + | - | - | - | - | 19 |
| 6 | U | 95 | + | + | + | + | + | + | + | + | - | - | - | - | + | + | + | + | + | + | + | - | - | + | - | + | - | + | - | - | + | - | + | 20 |
| 7 | U | 69 | + | + | + | + | + | + | - | + | - | - | - | - | + | + | + | - | - | + | + | - | - | - | + | - | - | - | - | - | - | - | - | 13 |
| 8 | U | 12 | + | + | + | + | + | + | + | + | - | - | - | - | + | + | + | + | + | + | + | + | - | + | - | - | + | + | - | - | - | - | - | 19 |
| 9 | U | 69 | + | + | + | + | + | + | - | + | - | - | - | - | + | + | + | - | - | + | + | - | - | - | + | - | - | - | + | - | - | - | - | 14 |
| 10 | U | 10 | + | + | + | - | - | - | - | - | - | - | - | - | - | + | + | - | - | + | + | - | - | - | + | - | - | - | - | - | - | - | - | 8 |
| 11 | U | 69 | + | + | + | + | + | + | - | + | - | - | - | - | + | + | + | - | - | + | + | - | - | - | + | - | - | - | - | - | - | - | - | 13 |
| 12 | U | 131 | + | + | + | + | + | + | + | - | - | - | - | - | + | + | + | + | - | + | + | - | + | + | - | - | - | - | - | - | + | - | - | 16 |
| 13 | U | 12 | + | + | + | + | + | + | + | + | - | - | - | - | + | + | + | + | - | + | + | + | - | + | - | + | + | + | + | - | - | + | - | 21 |
| 14 | U | 131 | + | + | + | + | + | + | + | + | - | - | - | - | + | + | + | - | - | + | + | - | - | + | + | - | + | - | - | - | - | - | - | 16 |
| 15 | U | 73 | + | + | + | + | + | + | + | - | + | - | - | - | + | + | - | + | - | + | + | + | - | + | - | + | + | + | + | + | - | - | - | 20 |
| 16 | U | 73 | + | + | + | + | + | + | + | + | + | - | - | - | + | + | + | + | + | + | + | + | - | + | + | + | + | + | + | + | - | - | - | 24 |
| 17 | U | 69 | + | + | + | + | + | + | - | + | - | - | - | - | + | + | + | + | - | + | + | - | - | - | + | - | - | - | - | - | - | - | - | 14 |
| 18 | U | 405 | + | + | + | + | + | + | + | + | - | - | - | - | + | + | + | - | - | + | + | - | - | - | + | - | + | - | - | - | - | - | - | 15 |
| 19 | U | 127 | + | + | + | + | + | + | + | - | - | + | - | - | + | + | - | + | - | + | + | + | - | + | - | + | + | + | + | + | - | - | - | 20 |
| 20 | U | 14 | + | + | + | + | + | + | + | + | - | - | - | - | + | + | + | - | + | + | + | - | - | + | + | + | + | - | - | - | - | - | - | 18 |
| 21 | U | 144 | + | + | + | + | + | + | + | + | - | - | - | - | + | + | + | - | + | + | + | + | - | + | + | + | + | - | - | - | - | - | - | 19 |
| 22 | U | 131 | + | + | + | + | + | + | + | + | - | - | - | - | + | + | + | - | - | + | - | - | - | + | + | - | - | - | - | - | - | + | - | 15 |
| 23 | U | 131 | + | + | + | + | + | + | + | + | - | - | - | - | + | + | + | - | - | + | + | - | - | + | + | - | - | - | - | - | - | + | - | 16 |
| 24 | O | 648 | - | + | + | - | - | - | - | - | - | - | - | - | + | + | + | - | - | + | - | - | - | - | - | - | - | - | - | - | - | - | - | 6 |
| 25 | O (E) | 4511 | + | + | - | - | - | - | - | - | - | - | - | - | - | - | - | - | - | + | - | - | - | - | - | - | - | - | - | - | - | - | + | 4 |
| 26 | O | 95 | + | + | - | + | + | + | + | + | - | - | - | - | + | + | - | - | + | + | + | + | - | + | - | + | - | + | - | - | - | - | + | 17 |
| 27 | O | 1882 | + | + | - | - | - | - | - | - | - | - | - | - | + | - | - | + | - | + | + | - | - | - | - | - | - | - | - | - | - | - | - | 6 |
| 28 | O | 7347 | + | - | - | - | - | - | - | - | - | - | - | - | - | - | - | - | - | + | + | - | - | - | - | - | - | - | - | - | + | - | - | 4 |
| 29 | O | 410 | + | + | - | - | - | - | - | - | - | - | - | - | - | - | - | - | - | + | - | - | - | - | - | - | - | - | - | - | + | - | - | 4 |
| 30 | O | Novel | + | + | - | - | - | - | - | - | - | - | - | - | + | + | - | - | - | + | - | - | + | - | - | - | - | - | - | - | - | - | - | 6 |
| 31 | O | 75 | + | + | + | + | + | + | + | - | + | - | - | - | + | - | - | + | + | + | - | - | - | - | - | - | + | - | + | + | - | - | - | 15 |
| 32 | O | 73 | + | + | + | + | + | + | + | + | - | - | - | - | + | + | + | - | - | + | + | + | - | + | + | + | - | + | - | + | - | - | - | 19 |
| 33 | O | 131 | + | + | + | + | + | + | + | - | - | - | - | - | + | + | + | + | - | + | + | - | + | + | - | - | + | - | + | - | + | - | - | 18 |
| 34 | O | Novel | + | + | - | - | - | - | - | - | - | - | - | - | + | - | - | + | - | + | - | - | - | - | - | + | - | - | - | - | - | - | - | 6 |
| 35 | O | 127 | + | + | + | + | + | + | + | - | - | + | - | - | + | + | - | + | - | + | + | + | - | + | - | + | + | + | + | - | - | - | - | 19 |
| 36 | O | 136 | + | + | - | - | - | - | - | - | - | - | - | - | + | + | - | + | - | + | - | - | + | - | - | + | - | - | - | + | - | - | - | 9 |
| 37 | O | 1643 | + | + | - | - | - | - | + | - | - | - | - | - | + | + | - | - | - | + | - | - | - | - | - | + | - | - | - | - | - | - | - | 7 |
| 38 | O | 127 | + | + | + | + | + | + | + | - | - | + | - | - | + | + | - | + | - | + | + | + | - | + | - | + | - | + | - | - | - | - | - | 17 |
| 39 | O | 216 | + | - | - | - | - | - | - | - | - | - | - | - | - | - | - | - | - | + | - | - | - | - | - | - | - | - | - | - | - | - | - | 2 |
| 40 | O | 648 | - | + | - | + | - | - | + | + | - | - | - | - | - | + | - | - | - | + | + | - | - | - | + | - | - | - | - | - | - | - | - | 8 |
| 41 | O | 88 | + | + | - | - | - | - | - | - | - | - | + | - | + | + | + | + | - | + | - | - | - | - | - | - | - | - | - | - | - | - | - | 8 |
| 42 | O | 127 | + | + | + | + | + | + | + | - | - | - | - | - | + | + | - | - | - | + | + | + | - | + | - | + | - | + | + | - | - | - | - | 16 |
| 43 | O | 73 | + | + | + | + | + | + | - | + | + | - | - | - | + | + | + | + | - | + | + | + | - | + | + | + | + | + | - | + | - | - | - | 21 |
| 44 | O | 131 | + | + | + | + | + | + | + | + | - | - | - | - | + | + | + | - | - | + | + | - | - | + | + | - | + | - | + | - | - | - | - | 17 |
| 45 | O | 399 | + | - | + | - | - | - | - | - | - | - | - | - | - | - | - | - | - | + | - | - | - | - | - | - | - | - | - | - | - | - | - | 3 |
| 46 | O | 131 | + | + | + | + | + | + | + | + | - | - | - | - | + | + | + | - | - | + | + | - | - | + | + | - | - | - | - | - | - | + | - | 16 |
| 47 | O | 648 | - | + | - | - | - | - | - | - | - | - | - | - | + | + | + | - | - | + | + | - | - | - | - | - | - | - | - | - | - | - | - | 6 |
| 48 | O | 73 | + | + | - | + | - | + | + | + | - | - | - | - | + | + | - | - | + | + | + | - | - | + | - | + | + | + | + | + | - | - | - | 17 |
| 49 | O | 1057 | + | + | + | + | + | + | + | + | + | - | - | - | + | + | + | + | + | + | + | - | - | + | + | + | + | + | + | - | - | - | - | 22 |
| 50 | O | 131 | + | + | - | - | - | - | - | - | - | - | - | - | + | + | - | + | - | + | + | - | + | + | - | - | - | - | - | - | + | - | - | 10 |
| 51 | O | 131 | + | + | + | - | - | - | - | + | - | - | - | + | + | + | + | - | - | + | + | - | - | + | + | - | - | - | - | - | - | - | - | 12 |
| 52 | N-T | 315 | - | + | - | - | - | - | + | - | - | - | - | - | - | - | - | - | - | + | + | - | - | - | + | + | - | - | - | - | - | - | - | 6 |
| 53 | N-T | 131 | + | + | + | - | - | - | - | + | - | - | - | - | + | + | + | - | - | + | + | - | - | + | + | - | - | - | - | - | - | - | - | 11 |
| 54 | N-T | 393 | + | + | + | + | + | + | - | + | - | - | - | - | + | + | + | - | - | + | + | - | - | - | + | - | - | - | - | - | - | - | - | 13 |
| 55 | N-T | 720 | + | + | - | - | - | - | + | - | - | - | - | - | - | - | - | - | - | + | + | - | - | - | - | - | - | - | - | - | + | - | - | 6 |
| 56 | N-T | 361 | + | - | - | - | - | - | - | - | - | - | - | - | + | - | - | - | - | + | - | - | - | - | - | - | - | - | - | - | - | - | - | 3 |
| 57 | N-T | 131 | + | + | + | - | - | - | - | + | - | - | - | + | + | + | + | - | - | + | + | - | - | + | + | - | - | - | - | - | - | - | - | 12 |
| 58 | N-T | 58 | + | + | - | - | - | - | - | - | - | - | - | - | + | + | + | + | - | + | - | - | - | - | - | - | - | - | - | - | - | - | - | 7 |
| 59 | N-T | 1170 | + | + | - | - | - | - | - | - | - | - | + | - | + | + | - | + | - | + | + | - | - | - | - | + | - | - | - | - | - | - | - | 9 |
| 60 | N-K | 73 | + | + | + | + | + | + | + | + | + | - | - | - | + | + | + | + | + | + | + | + | - | + | + | + | + | + | + | + | + | - | - | 25 |
| 61 | N-K | 69 | + | + | + | - | - | - | - | - | - | - | - | - | + | + | - | + | - | + | - | - | - | - | - | - | - | - | - | - | - | - | - | 7 |
| VF Prevalence across isolates (%) | | |  |  |  |  |  |  |  |  |  |  |  |  |  |  |  |  |  |  |  |  |  |  |  |  |  |  |  |  |  |  |  |  |
|  |  |  | 93 | 93 | 62 | 61 | 57 | 59 | 56 | 51 | 23 | 5 | 3 | 3 | 85 | 82 | 56 | 44 | 16 | 100 | 75 | 26 | 7 | 54 | 44 | 39 | 33 | 30 | 26 | 16 | 13 | 7 | 5 |  |

**Supplementary Table 2. *E. coli* bacteraemia isolate virulence factor gene and MLST data.** + virulence factor (VF) gene detected; - VF gene not detected. Focus of infection resulting in bacteraemia: U – urinary; O – non-urinary; O (E) – non-urinary and excluded from comparative VF analysis; N-T - neutropaenic unknown focus (translocation); N-K - neutropaenic, known focus and excluded from comparative VF gene analysis. Note: isolates 35 and 36 originated from same patient and represent two distinct infections separated by 46 days. All other isolates obtained from individual patients at a single time point.

| ***E. coli* isolate** | **Bacteraemia**  **source** | **MLST ST** | **Amoxicillin** | **Piperacillin** | **Co-amoxiclav** | **Piperacillin/**  **tazobactam** | **Cefuroxime** | **Cefotaxime** | **Ceftazidime** | **Ciprofloxacin** | **Gentamicin** | **Meropenem** | **Ertapenem** | **Chloramphenicol** | **Trimethroprim-**  **sulfamethoxazole** | **Colistin** | **Temocillin** | **ESBL (disc testing)** | **Genomic ESBL**  **(CTX-M type)** | **Antimicrobial**  **resistance score** | **MDR** | **Number of VF genes** |
| --- | --- | --- | --- | --- | --- | --- | --- | --- | --- | --- | --- | --- | --- | --- | --- | --- | --- | --- | --- | --- | --- | --- |
| 1 | U | 12 | S | S | S | S | S | S | S | S | S | S | S | S | S | S | S |  |  | 0 | N | 21 |
| 2 | U | 127 | S | S | S | S | S | S | S | S | S | S | S | S | S | S | S |  |  | 0 | N | 15 |
| 3 | U | 131 | **R** | **R** | **R** | S | **R** | **R** | **R** | **R** | **R** | S | S | S | **R** | S | S | Y | Y | 9 | Y | 12 |
| 4 | U | 131 | S | **R** | **R** | S | **R** | **R** | **R** | **R** | **R** | S | S | S | **R** | S | S | Y | Y | 8 | Y | 12 |
| 5 | U | 12 | S | S | S | S | S | S | S | S | S | S | S | S | S | S | S |  |  | 0 | N | 18 |
| 6 | U | 95 | S | S | S | S | S | S | S | S | S | S | S | S | S | S | S |  |  | 0 | N | 17 |
| 7 | U | 69 | S | S | S | S | S | S | S | S | S | S | S | S | S | S | S |  |  | 0 | N | 10 |
| 8 | U | 12 | **R** | S | S | S | S | S | S | S | S | S | S | S | S | S | S |  |  | 1 | N | 11 |
| 9 | U | 69 | S | S | S | S | S | S | S | S | S | S | S | S | S | S | S |  |  | 0 | N | 9 |
| 10 | U | 10 | **R** | **R** | S | S | S | S | S | S | S | S | S | S | S | S | S |  |  | 2 | N | 10 |
| 11 | U | 69 | S | S | S | S | S | S | S | S | S | S | S | S | S | S | S |  |  | 0 | N | 14 |
| 12 | U | 131 | **R** | **R** | S | S | S | S | S | S | S | S | S | S | S | S | S |  |  | 2 | N | 20 |
| 13 | U | 12 | **R** | **R** | S | S | S | S | S | S | S | S | S | **R** | **R** | S | S |  |  | 4 | Y | 14 |
| 14 | U | 131 | **R** | S | S | S | S | S | S | **R** | S | S | S | S | **R** | S | S |  |  | 3 | Y | 19 |
| 15 | U | 73 | **R** | **R** | **R** | **I** | S | S | S | S | S | S | S | S | **R** | S | S |  |  | 4.5 | Y | 22 |
| 16 | U | 73 | **R** | **R** | S | S | S | S | S | S | S | S | S | **R** | S | S | S |  |  | 3 | N | 11 |
| 17 | U | 69 | **R** | **R** | S | S | S | S | S | S | S | S | S | S | S | S | S |  |  | 2 | N | 13 |
| 18 | U | 405 | **R** | S | **R** | S | S | S | S | S | S | S | S | S | S | S | S |  |  | 2 | N | 20 |
| 19 | U | 127 | S | S | S | S | S | S | S | S | S | S | S | S | S | S | S |  |  | 0 | N | 16 |
| 20 | U | 14 | **R** | **R** | S | S | **R** | S | S | S | S | S | S | **R** | S | S | **R** |  |  | 5 | Y | 17 |
| 21 | U | 144 | **R** | **R** | S | S | S | S | S | S | S | S | S | S | S | S | S |  |  | 2 | N | 12 |
| 22 | U | 131 | **R** | **R** | **R** | I | **R** | **R** | **R** | **R** | **R** | S | S | **R** | **R** | S | **R** | Y | Y | 11.5 | Y | 13 |
| 23 | U | 131 | **R** | **R** | **R** | **I** | **R** | **R** | **R** | **R** | **R** | S | S | S | **R** | S | S | Y | Y | 9.5 | Y | 7 |
| 24 | O | 648 | **R** | **R** | **R** | S | **R** | S | S | **R** | S | S | S | **R** | **R** | S | S |  |  | 7 | Y | 18 |
| 25 | O (E) | 4511 | **R** | S | S | S | S | S | S | S | S | S | S | S | S | S | S |  |  | 1 | N | 4 |
| 26 | O | 95 | **R** | S | S | S | S | S | S | S | S | S | S | S | S | S | S |  |  | 1 | N | 14 |
| 27 | O | 1882 | **R** | **R** | S | S | S | S | S | S | S | S | S | **R** | **R** | S | S |  |  | 4 | Y | 6 |
| 28 | O | 7347 | S | S | S | S | S | S | S | S | S | S | S | S | S | S | S |  |  | 0 | N | 4 |
| 29 | O | 410 | S | S | S | S | S | S | S | S | S | S | S | S | S | S | S |  |  | 0 | N | 4 |
| 30 | O | Novel | S | S | S | S | S | S | S | S | S | S | S | S | S | S | S |  |  | 0 | N | 6 |
| 31 | O | 75 | **R** | **R** | **R** | **I** | **R** | **R** | **I** | S | S | S | S | S | S | S | S | N | N | 6 | Y | 14 |
| 32 | O | 73 | S | S | S | S | S | S | S | S | S | S | S | S | S | S | S |  |  | 0 | N | 16 |
| 33 | O | 131 | **R** | **R** | S | S | S | S | S | S | S | S | S | S | S | S | S |  |  | 2 | N | 17 |
| 34 | O | Novel | **R** | S | S | S | S | S | S | S | S | S | S | S | S | S | S |  |  | 1 | N | 6 |
| 35 | O | 127 | S | S | S | S | S | S | S | S | S | S | S | S | S | S | **R** |  |  | 1 | N | 19 |
| 36 | O | 136 | S | S | S | S | S | S | S | S | S | S | S | S | S | S | **R** |  |  | 1 | N | 9 |
| 37 | O | 1643 | S | S | S | S | S | S | S | S | S | S | S | S | S | S | S |  |  | 0 | N | 7 |
| 38 | O | 127 | S | S | S | S | S | S | S | S | S | S | S | S | S | S | S |  |  | 0 | N | 16 |
| 39 | O | 216 | S | S | S | S | S | S | S | S | S | S | S | S | S | S | S |  |  | 0 | N | 2 |
| 40 | O | 648 | S | S | S | S | S | S | S | **R** | S | S | S | S | S | S | S |  |  | 1 | N | 7 |
| 41 | O | 88 | **R** | S | S | S | S | S | S | S | S | S | S | S | S | S | S |  |  | 1 | N | 8 |
| 42 | O | 127 | **R** | S | **R** | S | S | S | S | S | S | S | S | S | S | S | S |  |  | 2 | N | 14 |
| 43 | O | 73 | S | S | S | S | S | S | S | S | S | S | S | S | S | S | S |  |  | 0 | N | 19 |
| 44 | O | 131 | **R** | **R** | S | S | **R** | **R** | S | **R** | **R** | S | S | S | **R** | S | S | N | Y | 7 | Y | 15 |
| 45 | O | 399 | S | S | S | S | S | S | S | S | S | S | S | S | S | S | S |  |  | 0 | N | 3 |
| 46 | O | 131 | **R** | **R** | **R** | **I** | **R** | **R** | **R** | **R** | **R** | S | S | **R** | **R** | S | S | Y | Y | 10.5 | Y | 13 |
| 47 | O | 648 | **R** | **R** | S | S | **R** | **R** | S | **R** | S | S | S | **R** | **R** | S | S | Y | Y | 7 | Y | 7 |
| 48 | O | 73 | **R** | **R** | S | S | S | S | S | S | S | S | S | S | S | S | S |  |  | 2 | N | 17 |
| 49 | O | 1057 | S | S | S | S | S | S | S | S | S | S | S | S | S | S | S |  |  | 0 | N | 20 |
| 50 | O | 131 | S | S | S | S | S | S | S | S | S | S | S | S | S | S | S |  |  | 0 | N | 10 |
| 51 | O | 131 | **R** | **R** | S | S | S | S | S | S | S | S | S | **R** | **R** | S | S |  |  | 4 | Y | 12 |
| 52 | N-T | 315 | **R** | **R** | **R** | **R** | **R** | S | S | **R** | **R** | S | S | S | **R** | S | S |  |  | 8 | Y | 6 |
| 53 | N-T | 131 | **R** | **R** | **R** | **R** | S | S | S | **R** | **R** | S | S | S | **R** | S | S |  |  | 7 | Y | 11 |
| 54 | N-T | 393 | **R** | **R** | S | S | **R** | S | S | **R** | S | S | S | **R** | S | S | **R** |  |  | 6 | Y | 10 |
| 55 | N-T | 720 | S | S | S | S | S | S | S | S | S | S | S | S | S | S | S |  |  | 0 | N | 6 |
| 56 | N-T | 361 | **R** | **R** | S | S | S | S | S | **R** | S | S | S | **R** | **R** | S | S |  |  | 5 | Y | 12 |
| 57 | N-T | 131 | S | S | S | S | S | S | S | **R** | S | S | S | S | S | S | S |  |  | 1 | N | 7 |
| 58 | N-T | 58 | S | S | S | S | S | S | S | S | S | S | S | S | **R** | S | S |  |  | 1 | N | 9 |
| 59 | N-T | 1170 | **R** | **R** | S | S | S | S | S | **R** | S | S | S | S | S | S | S |  |  | 3 | N | 7 |
| 60 | N-K | 73 | **R** | **R** | S | S | S | S | S | S | S | S | S | S | S | S | S |  |  | 2 | N | 3 |
| 61 | N-K | 69 | **R** | **R** | S | S | S | S | S | S | S | S | S | S | **R** | S | **R** |  |  | 4 | N | 23 |

**Supplementary Table 3. *E. coli* bacteraemia isolate antibiogram data.** Isolates 35 and 36 are from the same patient and represent two distinct infections separated by 46 days. All other isolates represent infiections in a single participant. Focus of infection resulting in bacteraemia: U - urinary ; O - non-urinary, O (E) - non-urinary and excluded from comparative virulence factor (VF) gene analysis; N-T - neutropaenic, unknown focus (translocation); N-K - neutropaenic, known focus and exlucded from comparative VF analysis. MLST - multi-locus sequence type; S - sensitive; R - resistant; I - intermediate; Y - yes; N - no; ESBL - extended-spectrum beta-lactamase; MDR - multi-drug resistant. MDR defined in line with international consensus guidelines, *i.e.* non-susceptible to ≧1 agent in ≧3 antimicrobial categories including aminoglycosides, anti-MRSA cephalosporins, anti-pseudomonal penicillins with betalactase inhibitors, carbapenems, non-extended spectrum cephalosporins (*i.e.* 1st and 2nd generation), extended-spectrum cephalosporins (*i.e.* 3rd and 4th generation), cephamycins, fluoroquinolones, trimethroprim-sulphamethoxazole, glycyclines, monobactams, penicillins, penicillins with betalactamase inhibitors, chloramphenicol, phosphonic acids and colistin.
